# A Gaussian Mixture Model-Based Continuous Boundary Detection for 3D Sensor Networks

**DOI:** 10.3390/s100807632

**Published:** 2010-08-13

**Authors:** Jiehui Chen, Mariam B. Salim, Mitsuji Matsumoto

**Affiliations:** 1 Global COE Program International Research and Education Center for Ambient SoC, Waseda University, Tokyo, 169-8555, Japan; 2 Graduate School of Global Information and Telecommunication Studies, Waseda University, Tokyo, 169-0051, Japan; E-Mails: msalim@fuji.waseda.jp (M.B.S.); mmatsumoto@waseda.jp (M.M.)

**Keywords:** 3D sensor network, Gaussian Mixture Model, continuous boundary detection

## Abstract

This paper proposes a high precision Gaussian Mixture Model-based novel Boundary Detection 3D (BD3D) scheme with reasonable implementation cost for 3D cases by selecting a minimum number of Boundary sensor Nodes (BNs) in continuous moving objects. It shows apparent advantages in that two classes of boundary and non-boundary sensor nodes can be efficiently classified using the model selection techniques for finite mixture models; furthermore, the set of sensor readings within each sensor node’s spatial neighbors is formulated using a Gaussian Mixture Model; different from DECOMO [1] and COBOM [2], we also formatted a BN Array with an additional own sensor reading to benefit selecting Event BNs (EBNs) and non-EBNs from the observations of BNs. In particular, we propose a Thick Section Model (TSM) to solve the problem of transition between 2D and 3D. It is verified by simulations that the BD3D 2D model outperforms DECOMO and COBOM in terms of average residual energy and the number of BNs selected, while the BD3D 3D model demonstrates sound performance even for sensor networks with low densities especially when the value of the sensor transmission range (*r*) is larger than the value of Section Thickness (*d*) in TSM. We have also rigorously proved its correctness for continuous geometric domains and full robustness for sensor networks over 3D terrains.

## 1. Introduction

Wireless sensor networks (WSNs) may consist of tiny, energy efficient sensor nodes communicating via wireless channels, performing distributed sensing and collaborative tasks for a variety of monitoring applications. One of the critical problems in sensor applications is detecting boundary sensors in a complex sensor network environment where sensed data is often required to be associated with spatial coordinates. In [2] a COBOM protocol that monitors the boundary of a continuous object was proposed. Sensor nodes are assigned with a Boundary sensor Node (BN) array to store BN information. The boundary monitoring is based on the changes to the observations in the BN array. As a updated version, [1] presented the DEMOCO protocol that enhanced COBOM by considering sensor nodes on one side of the boundary line called the “IN” range, and ignoring those on the other side of the boundary line called the “OUT” range which theoretically reduces approximately by half of the number of the selected BNs. Others like [3–6] also involve two-dimensional (2D) sensor localizations. To address the issues of adaptive sensor coverage and tracking for dynamic network topology, the authors of [7] utilized a Gaussian mixture model to characterize the mixture distribution of object locations and proposed a novel methodology to adaptively update sensor node placement according to the ML estimates of mass object locations with a distributed implementation of an EM algorithm to reduce communication costs. Moreover, [8] discussed a flocking-base mobility model for Distributed Kalman Filtering (DKF) in mobile sensor networks and [9,10] demonstrated efficient boundary detection algorithms with only the connectivity information.

In fact, the boundary detection problem has been mostly considered for 2D sensor networks and the case of 3D sensor networks has gone practically unnoticed. Despite the fact that difference between the normal 2D and the more realistic 3D scenario is only one extra dimension, network topology could be much more complex and the location scheme has to be more robust towards network irregularities. Taking a step further to expand from 2D to 3D sensor applications, several neighborhood-measurement [11] based 3D range-free boundary detection models [12–17] have been proposed. However, their tight dependence on sensor node densities and availability of sufficient neighbors are too optimistic for real 3D sensor applications due to their non-uniform sensor node densities and topology randomization. On the other hand, a range-based model such as in [18] does not make any assumption about sensor node densities and network topology. Instead it introduced a strong entity called mobile location assistants (LAs) that enables each location-unaware sensor node to easily estimate its own position using the measurable AOAs [11] and RSS [19]. Similar approaches like [5,6,20] assume that a small fraction of sensor nodes called anchors or beacons have a priori knowledge of their location and [21] proposed a range-based positioning method using beacon signals, that doesn’t require time synchronization since the beacon sensor nodes estimate the range based on frequency differences instead of time differences. To conclude, all the aforementioned approaches either introduced strong entities or made irrational assumptions. Furthermore, [22] presented a new high precision WSN positioning method with reasonable implementation cost for a 3D case. Reference sensor nodes with known locations transmit linear frequency modulation continuous waves (FMCWs), while other sensor nodes estimate the range difference to them based on the received signals' frequency difference, called time frequency difference arrival (TFDA).

Motivated by all above observations, instead of introducing miraculous assisting entities, our range-free Gaussian Mixture Model (GMM)-based approach performs a connectivity information-based segmentation algorithm [23] that partitions an irregular sensor field into nicely shaped pieces, associated with an enhanced BN Array and efficient distributed in-network information extraction virtual Thick Section Model (TSM); to the best of our knowledge, this is the first work that presents a principled algorithmic approach integrating computational geometry constructs adopted simultaneously for boundary detection in both 2D and 3D network areas. It is promising that our new statistical Gaussian mixture model [24]-based method in this paper is capable of fusing multivariate real-valued sensor inputs to detect boundaries of events in a mathematically principled manner. More precisely, the distribution of sensor readings within each sensor node’s spatial neighborhood is mathematically formulated using most popular finite GMMs. The model selection techniques [25–28] can then effectively identify the correct number of modes for finite mixture models. Therefore, Boundary and Non-Boundary sensor nodes can be consequently distinguished from their neighboring sensor node data distributions.

The remainder of this paper is organized as follows: the next section details enhancement to the BN Array concept; Section 3 simply describes general problems in boundary detection; Section 4 presents the proposed robust Boundary Detection scheme for 3D (BD3D) sensor networks in detail; Section 5 proves BD3D by simulation results; Finally, Section 6 concludes the paper with future work.

## 2. Enhancement to BN Array

**Figure 1.**
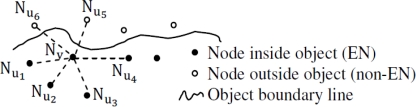
Readings of neighbors in BN Array of N_v_.

In [29] three different schemes which can only take inputs of the 0/1 decision predicates from neighboring sensor nodes are proposed. [30] presents a noise-tolerant algorithm named NED for event and event boundary detection. In NED, the moving mean of the readings of the neighboring sensor node set is used as the estimate for a certain sensor node. The authors of [31] propose Median-based approaches for outlying classification and event frontline detection, where the median is a useful and robust estimator which works directly with continuous numbers, rather than binary 0/1 readings. An extra description of the BN-Array of COBOM [2] and DECOMO [1] is given in this section. Suppose we have a sensor node v (N_v_) and its neighbors $\xi \left(N_{v}\right)=\sum_{i=0}^{k}N_{u_{i}}$ (k is the potential number of neighbors) (k = 6 in Figure 1). Let us consider the BN array in [1, 2]:

In Figure 1, the sensor readings of ξ(N_v_) only indicate the relative locations of its neighbors only. Correspondingly, there is no own sensor reading, as a result, N_v_ judges itself by inquiring ξ(N_v_) in a time and energy consuming way. In our model, we applied a head with 1 byte more space for the BN Array to store its own sensor reading as well (see Table 2) for self-judgment as a EBN or non-EBN. Here, we denote a BN inside object as Event BN (EBN), and a BN outside object as non-EBN. That is very important for monitoring applications in the sensor network because an Event sensor Node (EN) is usually highly responsible for sending and receiving the aggregated data should be constantly aware of own status.

**Figure 2.**
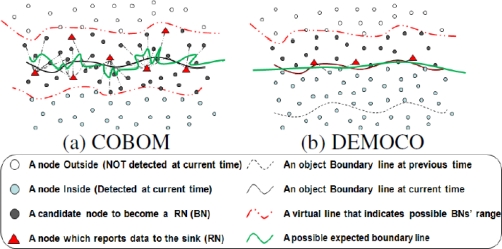
Expected boundary lines [1].

**Figure 3.**
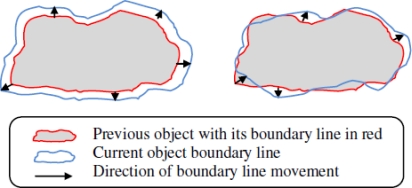
Possible boundary line changes when the object shrunk or expanded. **(a)** Regular variation boundary movement, **(b)** Irregular variation boundary movement.

Figure 2(a,b) show the expected boundary lines in COBOM and DEMOCO, respectively. Despite the fact that the shape of the expected boundary line in the 2D model of BD3D (see Figure 3) is similar to that of DEMOCO, the knowledge about Boundary sensor Nodes (BNs) promises to be different because we can clearly distinguish EBN and non-EBN as well.

## 3. Problem

We first present the problems before outlining how our proposal can benefit dynamic boundary detection for 2D and 3D sensor networks in the coming sections. To generally analyze the existing problems for superior boundary detection in a 3D impediment scenario, sensor nodes in the network usually have slight mobility which makes it difficult to establish their locations. Figure 3 illustrates two possible boundary line changes in a 2D scenario when the object is shrunk or expanded. Case (a) is relatively easy to manage, while (b) becomes a big problem that involves frequent inquiries among BNs and massive modifications to BN arrays.

## 4. Boundary Detection for a 3D Sensor Network (BD3D)

This section involves the main objective of achieving a flexible and energy-efficient 3D continuous boundary detection with a clear knowledge of EBN and non-EBN. Assume that sensor nodes are randomly deployed over 3-dimensional terrain. Each sensor node has limited resources (CPU, battery, etc), and is equipped with an omni-directional antenna. For the radio model, E_elec_ is for running the transmitter or receiver circuitry and *ɛ*_amp_ is for the transmit amplifier. To transmit a δ-bit message a distance *l* using this radio model, the radio expends (E_elec_ × δ + *ɛ*_amp_ × δ × *l*^2^), to receive the message, the radio expands (E_elec_ × δ) [32]. This energy model assumes a continuous energy consumption function. Moreover, we currently assume that sensor node failures are primarily caused by energy depletion. Note that in our model, no assumptions are made about (1) homogeneity of sensor node distribution; (2) network and BN density; (3) proximity of querying observers and sensor node synchronization.

Our major contribution could be creating a statistical property of the finite mixture model, especially the Gaussian mixture model (GMM) and adopting it to distributed sensing scenarios. Suppose that we have a set of data observations ψ_i_ = {χ_1_, χ_2_, ....*,* χ_n_}, n ≤ N (N is the total number of sensor nodes in the network) with each χ_i_ representing a D-dimensional random vector. Assume that ψ_i_ follows a k-component finite mixture distribution [24] as follows:

(1) $$𝒫(\chi_{i}|\theta )=\sum_{j=1}^{k}\alpha_{j}𝒫(\chi_{i}|\theta_{j}),j=1,2,...,k;I=1,2,...,n.$$

subject to $\sum_{j=1}^{k}$ α_j_=1

where α_j_ is the mixing weight or sometimes called the prior weight and θ_j_ is the set of parameters of the j^th^ mixture component 𝒫(χ_i_|θ). Denote θ = {α_1_, θ_1_, α_2_, θ_2_,...., α_k_, θ_k_}. The objective function of estimating θ from ψ_i_ is to maximize the log-likelihood criterion as follows:

(2) $$\text{Log}\prod_{i=1}^{N}\text{𝒫}\left(\psi_{i}|\theta \right)=\sum_{i=1}^{n}\text{log}\sum_{j=1}^{k}\alpha_{j}𝒫\left(\chi_{i}|\theta_{j}\right)$$

Therefore, the maximum likelihood estimator of θ is:

(3) $${\hat{\theta }}_{\text{ML}}=\;\text{arg}\;\underset{\theta }{\underset{︸}{\text{max}}}\left\{\text{log}\prod_{i=1}^{n}\text{𝒫}\left(\psi_{i}|\theta \right)\right\}$$

Obviously, θ̂_ML_ cannot be computed analytically from the above equation. Instead, GMM is applied as its general solver to iteratively find the maximum likelihood solution of θ̂_ML_. GMM is the most important class of finite mixture densities. GMM is formulated by using a Gaussian density 𝒢(χ_i_|μ_j_, ∑_j_) with its mean vector μ_i_ and covariance matrix Σ_j_ to replace the general probability density function 𝒢(χ_i_|θ_j_) in the finite mixture model:

(4) $$𝒫\left(\chi_{i}|\theta \right)=\Sigma_{j=1}^{k}\alpha_{j}𝒢\left(\chi_{i}|\mu_{j},\Sigma_{j}\right)$$

where a multi-dimensional multivariate Gaussian distribution is defined as:

(5) $$𝒢(\chi |\mu ,\Sigma )=\frac{1}{{\left|\sum \right|}^{\frac{1}{2}}{\left(2\pi \right)}^{\frac{D}{2}}}\text{exp}\left\{-\frac{1}{2}{\left(\chi -\mu \right)}^{\prime }\Sigma^{-1}(\chi -\mu )\right\}$$

The Bayesian Information Criterion (BIC) [33] is one of the most popular model selection criteria based on penalty terms of model complexity. In this paper, we use BIC for GMM model selection:

(6) $$\text{BIC}\;\left(\theta \right)=-2\text{log}\;\left(𝒫\left(\psi_{i}|\theta \right)\right)+\text{Klog}\;(m)$$

where, m is the data sample number, and K is the total number of parameters to be estimated in GMM.

In this paper, we provide an algorithm for classifying EBNs. Given a sensor network {S_i_}, we assume that sensor nodes are deployed with moderate density in the spatial terrain. From a mathematical perspective, sensor readings provide a dense, but discrete sampling of the underlying continuous distribution. To check whether or not N_v_ is a sensor node lying on the boundary of an event, we put the data {χ_n_} from readings of the sensor nodes in ξ(N_v_) and then build our best GMM based on {χ_n_}.

In more detail, we first set the upper bound of the mixture component number to be K. Then for each j = 1,2,…,K, the data set {χ_n_}is fed into (5) (6) for estimation of θ(J). Let BM denote the number of mixture components of the best model. We select BM where $\text{BIC}\;\left(\theta \left(\text{BM}\right)\right)={\text{min}}_{J=1}^{K}\text{BIC}\;(\theta \left(J\right))$. Therefore our final is θ(BM) or {μ_j_, Σ_j_, α_j_}_j=1,2,....,BM_.

To classify if N_v_ is a EBN, the conditional probability for χ_i_ given model θ′(BM) is computed by

(7) $$𝒫\left(\chi_{i}|\theta^{\prime }\left(\text{BM}\right)\right)=\Sigma_{j=1}^{\text{BM}}\alpha_{j}𝒢\left(\chi_{i}|\mu_{j},\Sigma_{j}\right)$$

then 𝒫(χ_i_|θ′(BM))< γ, N_v_ is classified as a EBN. Where γ is used as a threshold to measure EBN which has significantly low probability density values given the final model θ′(BM). The threshold is set as γ = 0.25, the upper bound of the component number is set as K = 5. These parameters are used as the default in Sections 4 and 5, unless otherwise stated.

To dynamically update the estimates of observations by conducting [33], we have the following dynamic evolvement and observation equations:

(8) $$\chi_{v}^{i}=f(\chi_{v}^{i-1})+w_{v}^{i}$$

(9) $$\chi_{v}^{i}=g(\chi_{v}^{i-1})+v_{v}^{i}$$

where *f*(·) is the linear or nonlinear state evolvement function and *g*(·) is highly nonlinear observation function. $w_{v}^{i}$ and $v_{v}^{i}$ are the standard deviation (noise sequences). For example in static sensor node location, where $\chi_{v}^{i}$ remain the same after deployment because of the governance of Equation (8). Therefore, we get the expression:

(10) $$\chi_{v}^{i+1}=f(\chi_{v}^{i})+w_{v}^{i}$$

where, $w_{v}^{i}$ model the small position perturbation or other effects.

### 4.1. BD3D scheme in 2D model [D = 2 in (5)]

Suppose *n* total sensor nodes are randomly distributed in a 2D terrain, with network density μ enough to perform a boundary detection application. BD3D provides simultaneous selection of EBN and non-EBN during BN selection process by tactfully using the proposed BD3D BN Array (see Table 3) and GMM-based mathematic model.

Although the determination of sensor node status e.g., EBN or non-EBN *etc.* is practically meaningful, in literature fewer works are focusing on this issue. In BD3D, we tactfully utilize a BD3D BN array to adequately energy-friendly determine sensor node status (see Table 4).

$$\begin{array}{c} \text{head}=\{\begin{array}{cc} 1 & \text{EN},\text{EBN}\left(\text{BN}\right),\text{non}-\text{BN}\\ \;0 & \text{non}-\text{EN},\;\text{non}-\text{BN} \end{array}\\ \text{HR}=\{\begin{array}{cc} \text{all}\;1 & \text{non}-\text{EBN}\left(\text{BN}\right),\text{non}-\text{BN}\\ \;\text{all}\;0 & \text{EBN}\left(\text{BN}\right),\text{non}-\text{BN}\\ \text{random} & \text{EN},\text{non}-\text{EN or EBN}\left(\text{BN}\right) \end{array} \end{array}$$

$N_{v}^{i}$ is determined to be EN or EBN (BN) if head is 1 and HR is random based on the values in Table 5.

Expanding from 2D to 3D, we find that not only the sensing area of sensor node but the network topology is getting more complex, therefore when talking about the relative position of sensor nodes, we need 3D sense of space to construct the model.

### 4.2. BD3D 3D model [D = 3 in (5)]

**Figure 5.**
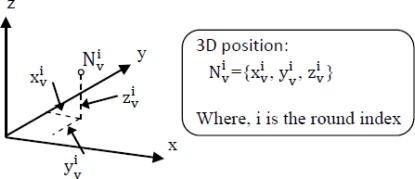
Position of N_v_ in 3D co-ordinate.

Define the state variable as 3D position for a specific sensor node modeled in Figure 5.

**Figure 6.**
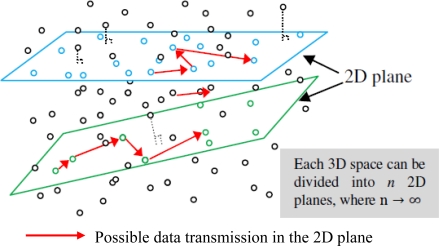
Concept of 2D plane for 3D sensing space.

**Figure 7.**
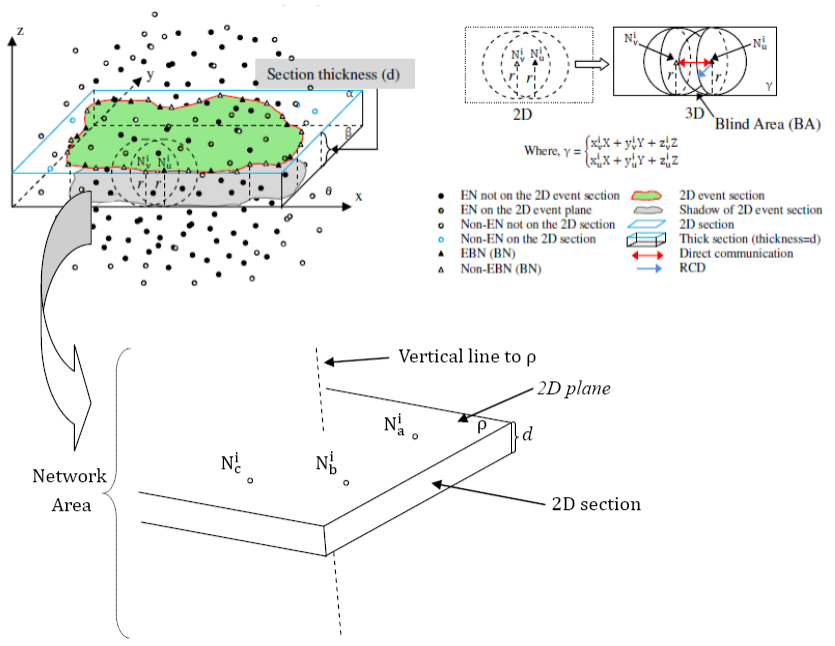
TSM concept with *d* = *r* for explanation simplicity.

In a 3D sensing space, sensor nodes are randomly distributed to form a network. To simplify the complicated operations in dealing with sensor node localization in 3D model, we apply a new concept of 2D plane that each 3D space can be divided into *n* 2D planes, where *n* → ∞(see Figure 6). The methodology of selection and representation of the 2D plane is described as:

- Randomly pick up to three sensor nodes { $N_{a}^{i}\left(x_{a}^{i},y_{a}^{i},z_{a}^{i}\right)$, $N_{b}^{i}\left(x_{b}^{i},y_{b}^{i},z_{b}^{i}\right)$, $N_{c}^{i}\left(x_{c}^{i},y_{c}^{i},z_{c}^{i}\right)$} (see Figure 7) from the 3D sensing space to form a 2D plane (either the 2D plane in blue or green in Figure 6).
- Suppose $N_{a}^{i}$ $N_{b}^{i}$ and $N_{c}^{i}$ are arbitrary points (sensor nodes) on the formed 2D plane called “ρ”, the plane representation is (see Figure 7).

(11) $$\rho =\{\begin{array}{l} x_{a}^{i}X+y_{a}^{i}Y+z_{a}^{i}Z\\ x_{b}^{i}X+y_{b}^{i}Y+z_{b}^{i}Z\\ x_{c}^{i}X+y_{c}^{i}Y+z_{c}^{i}Z \end{array}$$

Strictly speaking, a 2D plane is definitely as a 2D section (see Figure 7). Therefore, one 3D sensor network needs n 2D sections (*n* → ∞) to reconstruct. Due to the impossibility of computing *n* in programming, we introduce a virtual Thick Section Model (TSM).

Figure 7 may help understand the concept of TSM. A 2D section is modeled as a thick plane (ρ) with section thickness (*d*) with a set of representative points { $N_{a}^{i}\left(x_{a}^{i},y_{a}^{i},z_{a}^{i}\right)$, $N_{b}^{i}\left(x_{b}^{i},y_{b}^{i},z_{b}^{i}\right)$, $N_{c}^{i}\left(x_{c}^{i},y_{c}^{i},z_{c}^{i}\right)$} describing the elements of the section. In our model the boundaries are actually modeled as parametric line segments and points taking into account not only the position of the plane but also the uncertainties of the plane contour.

**Figure 8.**
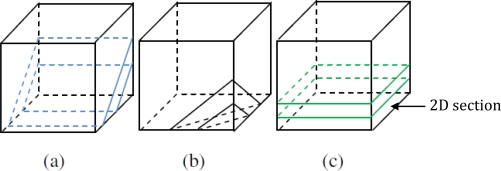
Possible 2D sections in 3D network area and (c) is the model used in simulations.

Suppose the 3D network area is ζ^3^ cube (ζ is pre-determined in programming) and *d* ≠ *r*. Therefore the selected 2D section could be those in Figure 8:

The 2D section in our simulation model actually is a (ζ^2^*d) area [see Figure 8 (c)]. (the section thickness (*d*) is determined a priori to programming).Thus, the simulator only need to perform TSM (ζ/*d* times that significantly improve the maneuverability.

## 5. Simulation

In this section, we evaluated the performance of BD3D 2D and 3D model implemented in Matlab respectively. The simulation parameters are given in the following table:

**Table:** 

**Parameter**	**Value**
Network Area (2D,3D)	(100 m)^2^(100 m)^3^
Number of sensor nodes(2D,3D)	2,500,10,000
The sink (2D,3D)	(50,175), (50,175,50)
Transmission range(2D)	10 m
Time slots	100 seconds
Initial Energy	2J/battery
Message size	100 Bytes
E_elec_	50 nJ/bit
E_fs_	10 pJ/bit/m2
δ_amp_	0.0013 pJ/bit/m4
E_DA_	5 nJ/bit/signal

Sensor nodes make local observations every 2 time slots

### 5.1. Simulation model

(2D model)

**Figure 9.**
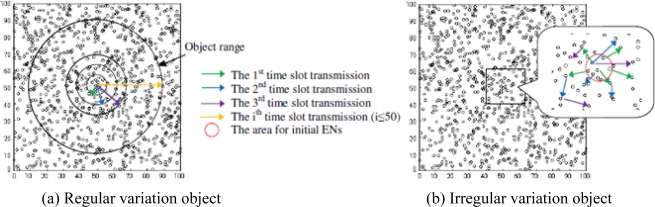
Sample of BD3D 2D model with regular variation and irregular variation object.

- Design a regular variation object: a circle initially centered at (50, 50) and continually expand it by increasing its radius by 10 meters every 10 time slots. (see Figure 9.)
- Design an irregular variation object: the initial ENs that adequately covers a circle area {(x − 50)2+(y−50)2 = Rcircle2} to initiate the event. At every time slot, EN propagates by picking up a random number of neighbors to join the event (non-EN→EN). In this way, the network is guaranteed to be fully connected. (see Figure 9.)

(3D model)

- Design a regular variation 3D object: the object center is (50, 50, 50) and continually expand its radius by 10 meters every 10 time slots.
- Design an irregular variation 3D object: the initial ENs are within a spherical area {(x − 50)^2^+(y − 50)^2^+(z − 50)^2^ = R_sphere_^2^. EN propagates in a similar way as that used for the irregular variation object in 2D model.

The BD3D is flexible enough to be used in a clustered network or a non-clustered network since it does not put any constraints on cluster architecture. However, BNs are usually heavily utilized to send aggregated data associated with the object/network boundary information to cluster head (in clustered networks) or the sink (non-clustered networks), they would run out of energy more quickly. Therefore, achieving a reasonable amount of BNs (the less the better) benefits energy saving.

### 5.2. BD3D 2D model

**Figure 11.**
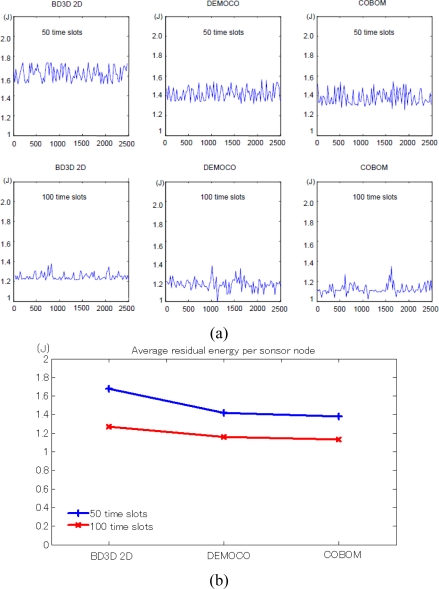
Average energy level status of 2,500 sensor nodes after 50 and 100 time slots operation.

This section discusses the performance evaluations based on BD3D 2D model. Figure 11 demonstrates the performances of the BD3D 2D model and DEMOCO and COBOM in terms of the average residual energy per sensor node at 50 and 100 time slots of operation respectively. Obviously, the performance of BD3D 2D is apparently better than DEMOCO and COBOM. However at the meanwhile, it shows the good stability of energy load balancing among the sensor nodes over the individual residual energy differences.

**Figure 12.**
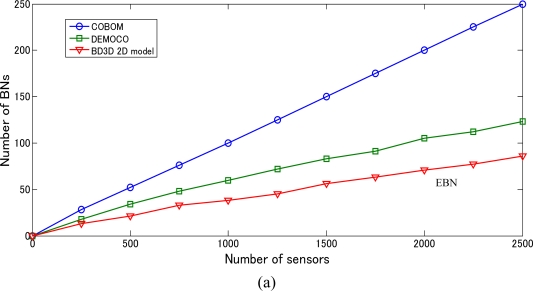

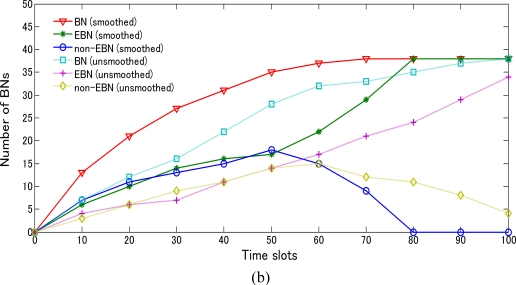
Performance evaluation by using BD3D 2D when *r* = 10 m. **(a)** Comparison with DEMOCO and COBOM on the number of BNs based on the number of sensor nodes (regular variation object case for 50 time slots) **(b)** Number of BNs (EBNs and non-EBNs) based on time slots (both regular variation and irregular variation object case).

Comparison of the number of BNs for a regular variation object with COBOM and DEMOCO is shown in Figure 12 (a). To increase the comparability, the network is only operated during 50 time slots. Figure 12 (a) shows that the BD3D 2D model consistently provides less than half of the BNs selected by COBOM and reduces approximately by 1/3 those achieved by DEMOCO in the same environment. This could be due to the use of the BD3D BN array (see Section 2) and GMM that helps selecting potential BNs easier than the aforementioned COBOM and DEMOCO. Consequently, this avoids low data delivery rates and excessive energy consumption by frequent flooding of inquiring packets

However, due to the elusive ways proposed to expand the irregular variation object, we can hardly do comparison with COBOM and DEMOCO anymore. Figure 12 (b) shows only the performance evaluation of the BD3D 2D model. As promised, EBNs and non-EBNs for regular variation and irregular variation object cases are clearly found. From our analysis, the value of BN (irregular variation) tends to be affected by irregular BL movements due to the elusive change of object compared to that of BN (regular variation) that looks more euphemistic. When the regular variation object expands over the *rebound distance* which indicates the end of saturated distribution around the BL, Figure 12 (b) shows a rapid increase of EBN (regular variation) and decrease of non-EBN (regular variation) until the object covers the whole network. On the other hand, due to the non-determinacy of irregular variation object shape changes, trajectories of both EBN (irregular variation) and non-EBN (irregular variation) are always difficult to size up. However, we find similarity or resemblance as to be essentially interchangeable for the first 50 time slots of operation between regular variation and irregular variation object cases. For the second 50 time slots, the performance of EBN (irregular variation) and non-EBN (irregular variation) are going to split up, but show no direct relationship with *rebound distance* and *boundary distance*.

### 5.3. BD3D 3D model

**Figure 13.**
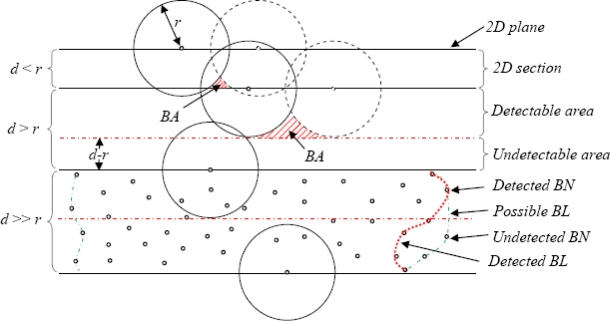
A combinational vertical section view of 3D sensor network with {*d* < *r*, *d* > *r*, *d* >> *r*}.

Figure 13 shows a vertical section view of 3D sensor network area using TSM—a combinational view of three conditions { *d < r, d > r, d >> r* }

**Figure 14.**
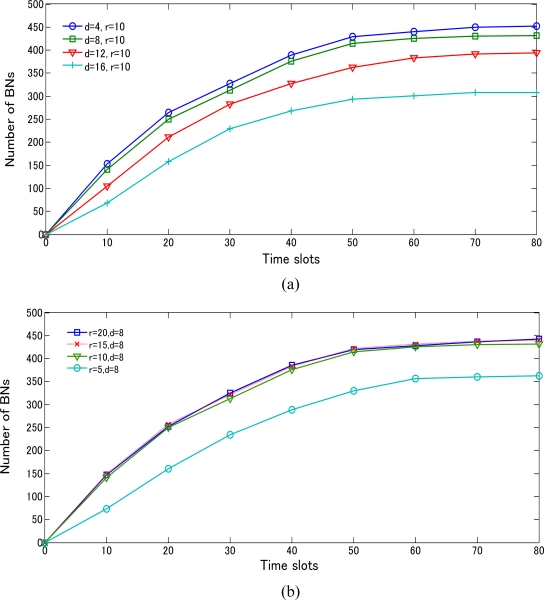
Comparison for regular variation object case using BD3D 3D model. **(a)** Number of BNs based on time slots via varying *d* (*r* = 10 m), **(b)** Number of BNs based on time slots via varying *r* (*d* = 8 m).

In this section, we modeled the BD3D 3D with different values of *r* and *d* by using TSM for regular variation and irregular variation objects, respectively. Figure 14(a) compares the number of BNs based on the value of *d* with *r* = 10m. As *d* < *r*, the values we got are approximately the same. Moreover, we varied the value of *d* (*d* > *r*) for simulating the cases with the significant existence of BA (see Figure 13), many BNs of highly possible BA got lost, resulting in decrease of the number of BNs. Figure 14(b) compares the number of BNs based on the value of *r* with *d* fixed at 8 m. As *d* > *r*, the performance shows the comparatively worst. By increasing *r* to meet *d* < *r*, it shows the significant improvement on the performances with very imperceptible distinctions. It is easy to guess that if communication range of a sensor node is large, there will be many neighbors that it can communicate with, which will result in more BNs.

**Figure 15.**
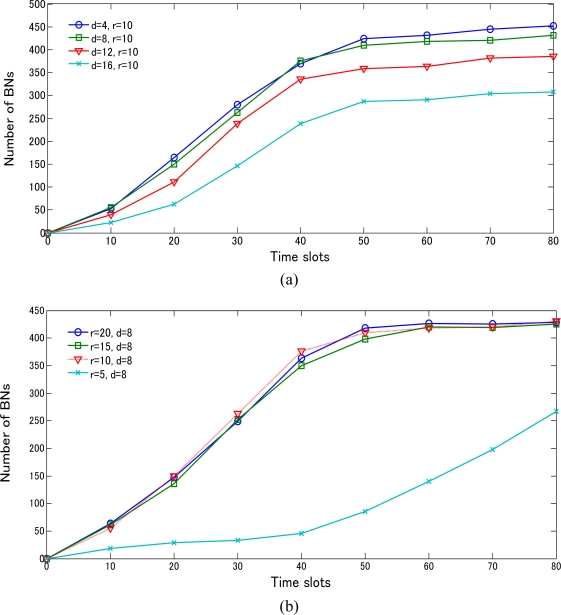
Performance comparison for irregular variation object case using BD3D 3D model. **(a)** Number of BNs based on time slots via varying *d* (*r* = 10m); **(b)** Number of BNs based on time slots via varying *r* (*d* = 8m).

Meanwhile, we set the same parameter environment in the BD3D 3D model for evaluating the number of BNs in the network in Figure 15. The most interesting feature is that the network based on *d* < *r* apparently performs better than that with *d* > *r*. As a result, it can be alleged that there is no strong relationship between the number of BNs and the communication range (*r*) using TSM once *d* < *r*. This occurrence can be clarified by the analysis illustrated in Figure 13. Another interesting feature we can observe from Figure 15(b) is that when *r* = 5 and *d* = 8, it undergoes a very slow increase first and then experiences a sharp increase in the number of BNs after 40 time slots. This was caused by a phenomenon that the objects expand highly depending on the number of existing BNs. However, at network initialization, we have relatively fewer existing BNs. As the cardinal number designating the existence of BNs is over a special value (available at around 40 time slots), the performance miraculously achieves a sudden improvement.

We hereby conclude that our BD3D for continuous boundary detection in 3D case works well especially when *d* < *r* using TSM. An in depth study about the impact of localization impact on various routing protocols and its implications on design of location-dependent system are left as future work.

## 6. Conclusions

This paper has proposed a novel Gaussian Mixture Model-based BD3D scheme for boundary detection of continuously moving object in a 3D sensor network. We adequately presented the proposed protocol, and the simulation results shown support our allegation that the BD3D 2D model surely outperforms COBOM and DEMOCO in terms of average residual energy per sensor node and the number of selected BNs, and the BD3D 3D model achieves accurate boundary detections by soundly selecting EBN and non-EBN for both regular variation and irregular variation object cases. Our future work will include additional optimization desired to improve the performance of our algorithm and verification of the precision of the expected boundaries and invention of a new protocol that considers data losses and route failures due to unpredictable errors such as sensor node failures, contention, interference and fading [35,36]. Moreover, the more accurate energy and mobility model will be addressed in future work.

## Figures and Tables

**Figure 4.**
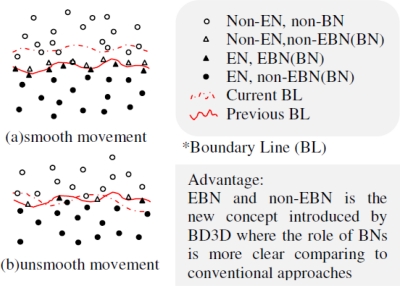
EBN and non-EBN on BL in BD3D 2D model when object expanded or shrunk.

**Figure 10.**
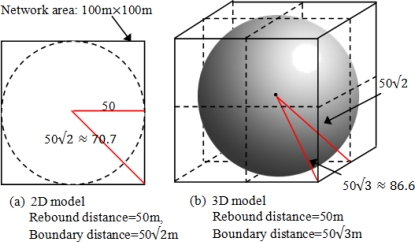
Rebound and boundary distances for BD3D.

**Table 1.**
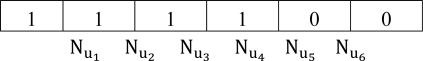
BN Array of N_v_ [Note: “0” and “1” are sensor readings (sample)].

**Table 2.**
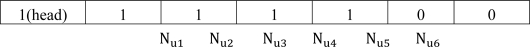
BD3D BN Array of N**_v_**.

**Table 3.** BD3D BN Array of N_v_.

Sensor reading of N_v_ (head)	Sensor readings of ξ(N_v_)(rear)

Note: $\xi \left(N_{v}\right)=\sum_{i=1}^{k}N_{u_{i}}$ (see Section 2) and both head and rear are initialized with “0”; Rule: a sensor node is EN if its own reading equals to “1” and vice versa.

**Table 4.** Head &HR based sensor node status determination.

**BD3D BN Array**	$N_{v}^{i}$					
	*EN*	*Non-EN*	*BN*		*Non-BN*	
			*EBN*	*Non-EBN*		
Head	1	0	1	1	0	1
HR	random	random	All 0 & random	All 1	All 0	All 1

* EBN ∈ EN, EBN ∪ non-EBN = BN (see Figure 4) and *random* means it is either all 1 or all 0.

**Table 5.** Example of BD3D BN Array of $N_{v}^{i}$.

1	0	1	1	1	1	1	0	1
